# Bacterial blood microbiome of *Mastomys* rodents: implications for disease spill-over at the animal-human interface within the Bushbuckridge-East community, South Africa

**DOI:** 10.3389/fcimb.2025.1520086

**Published:** 2025-02-03

**Authors:** Agatha O. Kolo, Kelly A. Brayton, Nicola E. Collins, Armanda D. S. Bastos, Sonja Matthee, Cory A. Gall, Jeanette Wentzel, Luis Neves, Marinda C. Oosthuizen

**Affiliations:** ^1^ Department of Veterinary Tropical Diseases, University of Pretoria, Pretoria, South Africa; ^2^ Department of Molecular Microbiology and Immunology, The University of Texas at San Antonio, San Antonio, TX, United States; ^3^ Department of Veterinary Microbiology and Pathology, Washington State University, Pullman, WA, United States; ^4^ Department of Zoology and Entomology, Mammal Research Institute, University of Pretoria, Pretoria, South Africa; ^5^ Hans Hoheisen Research Centre, Department of Veterinary Tropical Diseases, Faculty of Veterinary Science, University of Pretoria, Pretoria, South Africa; ^6^ Department of Conservation Ecology and Entomology, Stellenbosch University, Matieland, South Africa; ^7^ Centro de Biotecnologia, Eduardo Mondlane University, Maputo, Mozambique

**Keywords:** Bartonella, Ehrlichia, Anaplasma, Coxiella burnetii, 16S rRNA gene, gltA gene

## Abstract

The Bushbuckridge-East community in Mpumalanga Province, South Africa is bordered by nature reserves, including the Manyeleti Game Reserve. Murid rodents are prevalent in both Manyeleti and communal rangelands adjoining the community households. Although rodents are reservoir hosts for a broad range of viral, bacterial and parasitic pathogens, the rodent microbial diversity and transmission of zoonotic agents to humans in the community is understudied. In this study we investigated bacterial diversity in wild and commensal rodents sampled from different habitats. The 16S rRNA gene was amplified from DNA extracted from the blood of 24 wild *Mastomys* and one *Steatomys* sp. and subjected to PacBio circular consensus sequencing. As *Bartonella* species were dominant in the blood microbiome, *gltA* gene characterization was performed to delineate species. Rodents sampled from peri-urban and communal rangelands had higher proportions of *Bartonella* spp. [Hlalakahle (77.7%), Gottenburg (47.8%), Tlhavekisa (83.8%)] compared to those from the protected habitat (43.8%). *Ehrlichia* spp., *Anaplasma* spp., and *Coxiella burnetii* were detected at <1% of the sequence reads. Conventional PCR and sequencing validated the detection of *Bartonella* spp. with the first confirmation of *Bartonella mastomydis* infection in *Mastomys* in South Africa. Additionally, 317 mites, 90 fleas, 10 ticks and eight lice were collected from the rodents, providing evidence of possible vectors of the organisms detected. The detection of zoonotic agents in rodents in Bushbuckridge-East community, together with prior serological confirmation of *Bartonella* and *Coxiella* in non-malarial acute febrile patients from this community, highlights the possible risks that commensal rodents pose to human health.

## 1 Introduction

Rodents are reservoirs for over 60 zoonotic pathogens, contributing to the global emergence and re-emergence of infectious diseases (Woolhouse and Gowtage-Sequeria, 2005; Luis et al., 2013). Representing 43% of mammalian diversity, rodents are integral to the animal-human interface in Bushbuckridge-East, South Africa, where their prevalence in homes reaches 76% (Huchon et al., 2002; Berrian et al., 2016). Studies in this area have connected rodent-borne zoonoses with acute febrile illnesses in humans, finding 9.5% of such patients positive for *Bartonella* spp. and showing exposure to *Coxiella burnetii* and *Leptospira* spp. in others (Simpson et al., 2018). Given the association of certain *Bartonella* species with rodents, monitoring these hosts is vital, because their role in transmitting pathogens in Bushbuckridge-East remains unclear. Prior research in the area identified *Anaplasma phagocytophilum* in *Mastomys natalensis* and *Rattus tanezumi* captured from urban and periurban areas using 16S rRNA and *gltA* gene sequencing, highlighting the need for continued surveillance (Kolo et al., 2020).

*Mastomys natalensis* and *R. tanezumi*, closely associated with humans, may transmit infections (Skinner and Chimimba, 2005; Bonwitt et al., 2017). Next-generation sequencing (NGS) has unveiled diverse bacterial communities across hosts (Greay et al., 2018), yet rodent microbiomes remain underexplored (Cohen et al., 2015; Razzauti et al., 2015; Rynkiewicz et al., 2015; Ge et al., 2018). Research in China and France focused on bacteria detected from rodent spleens (Razzauti et al., 2015; Ge et al., 2018), while studies in Israel and the US examined the microbiome of rodent blood and their ectoparasites (Cohen et al., 2015; Rynkiewicz et al., 2015). These studies emphasized the importance of understanding host and vector bacterial communities as tools for managing vector-borne diseases (Cohen et al., 2015; Rynkiewicz et al., 2015). Despite rodents’ role as reservoirs for tick-borne pathogens (Telfer et al., 2007), South Africa’s wild murid rodents’ pathogenic diversity is poorly documented, often limited to single-genus studies (Hatyoka et al., 2019a, 2019; Kolo et al., 2020).

*Mastomys natalensis and M. coucha*, morphologically similar multimammate mice, are prevalent in southern Africa’s natural and human environments (Skinner and Chimimba, 2005), inhabiting grain stores and homes (De Graaff, 1981). *Mastomys coucha* is the primary host for flea-borne *Yersinia pestis* the cause of bubonic plague (Davis, 1964), while *M. natalensis* is associated with the Lassa fever virus in West Africa (Skinner and Chimimba, 2005).

The aim of this project was to elucidate the bacterial pathogens present in the blood of *Mastomys* species captured from different habitats at the animal-human interface in the Bushbuckridge-East community by sequencing of near full-length 16S rRNA gene amplicons.

## 2 Materials and methods

### 2.1 Ethics approval

The study received approval from the University of Pretoria’s Faculty of Veterinary Science animal ethics committee (V105-15). Research permissions for trapping and transporting rodents were granted by the South Africa Department of Agriculture, Land Reform and Rural Development (DALRRD) (12/11/1/1, 12/11/1/1/6), and the Mpumalanga Tourism and Parks Agency (MTPA, B1/290/2016), in accordance with the Animal Diseases Act (Section 20, 1984).

### 2.2 Study area and sample collection

The research was conducted at the human-livestock-wildlife interface in Bushbuckridge bordering Manyeleti Game Reserve. A total of 282 rodents were captured across urban/periurban (Gottenburg and Hlalakahle), rangeland (Tlhavekisa), and protected areas (Manyeleti) from 2014-2015 (**Supplementary Figure S1**). Morphological identification (Stuart and Stuart, 2001) and sex was recorded. Rodents were humanely euthanized using isoflurane, and blood was collected via cardiac puncture in EDTA tubes and on FTA ™ cards at the Hans Hoheisen Wildlife Research Station. Blood samples were transferred to the University of Pretoria’s BSL3 lab where DNA was isolated using a QIAamp^®^ DNA Mini Kit and stored at -20°C. The study utilized 24 samples from *Mastomys* spp., and one *Steatomys* sample. Seven samples were from Gottenburg, while the other sites had six samples each.

### 2.3 Molecular typing of *Mastomys* species

*Mastomys* rodents, a cryptic species complex, require molecular methods for accurate identification (Bastos et al., 2005). Two mitochondrial regions: the cytochrome *b* (*cytb*) gene and the barcoding region of cytochrome c oxidase subunit 1 (COI) were used for identification. Amplification and sequencing were performed at the University of Pretoria and Genelethu labs (Johannesburg), with COI sequences deposited and accessible through the portal of the Barcode of Life database (BOLD) https://portal.boldsystems.org/ with record set code (ZTBP) and in Genbank with accession numbers (PQ814604-PQ814628). *Cytb* sequences were deposited in Genbank with accession numbers (PP790712-PP790735).

### 2.4 Collection of ectoparasites

Regulations of the South African Department of Agriculture, Land Reform and Rural Development, (DALRRD) required that whole body rodent carcasses must first be frozen at -80°C and stored at Hans Hoheisen Research Centre, Mpumalanga South Africa before transport to a BSL3 provincial laboratory at Stellenbosch, South Africa. Ectoparasites, including ticks, fleas, lice, and mites, were removed in the BSL3 laboratory and preserved in absolute ethanol for morphological identification, using established taxonomic information (Till, 1963; Tipton et al., 1966; Ledger, 1980; Segerman, 1995; Walker, 2000; Horak et al., 2018). All ticks and lice were identified. A subsample of mites (trombiculid and mesostigmatan) and only male fleas were identified.

### 2.5 PCR amplification and sequencing

The 16S rRNA gene (V1-V8 variable regions) was amplified from 25 rodent samples with primers 27F and 1435R (**Supplementary Table S1**), as described (Gall et al., 2016). PCR was conducted using barcoded primers (**Supplementary Table S2**), Phusion Flash^®^ High Fidelity PCR Master Mix, and 100 ng of DNA, with three technical replicates (Gall et al., 2016). *Anaplasma centrale* DNA and PCR grade water were used as PCR controls. Cycling parameters included 98°C for 30s, 35 cycles at 98°C for 10s, 60°C for 30s, 72°C for 30s, and a final extension at 72°C for 10 min. PCR products, visualized on a 1.5% agarose gel were purified with the QIAquickPCR^®^ purification kit, and sent to Washington State University for Circular Consensus Sequencing on the PacBio platform.

### 2.6 16S rRNA sequence analysis

Sequence data was processed using PacBio software, adhering to predefined size and precision parameters. Genus-level classification of reads utilized the RDP 16S classifier (Cole et al., 2009) and NCBI BLASTn analysis for sequence identification. BLASTn results were filtered using Microsoft Excel, using a 1275 bp length and 98% identity threshold (Gall et al., 2016). Sequences below this threshold were classified at the genus level, while higher matches were identified to the species level (Jones et al., 2010; Bonnet et al., 2014; Budachetri et al., 2014). Operational taxonomic units (OTUs) representing less than 1% of total sequences were categorized as ‘rare’ (Gall et al., 2016). Raw microbiome sequence data were deposited in the NCBI sequence read archive (SRA) with accession numbers SRX5967121-SRX5967145 (**Supplementary Table S3**, **Supplementary Material**). For *Bartonella*, 16S consensus sequences were extracted using CLC genomics workbench 9.5.1 (Qiagen) and aligned with GenBank entries. The Jmodel test 1.3 predicted GTR + I + G as the optimal model (Darriba et al., 2012) selected under the Bayesian information criterion (BIC), and phylogenetic analysis was conducted using the maximum likelihood method in MEGA 11 (Tamura et al., 2021).

Rodent blood microbial compositions were analyzed with the community ecology package vegan 2.5-2 (Oksanen et al., 2016) in R studio (R Core Team, 2013). Alpha diversity was assessed through rarefaction curves, determining mean bacterial species diversity across habitats. Principal component analysis (PCA) quantifying bacterial population similarities in rodent blood, was done using FactoMineR (Lê et al., 2008). Similar blood bacterial profiles resulted in clustering, while dispersion showed variability. The variables proximity on the PCA plane suggested positive correlations; while opposite positioned variables indicated negative correlations. Correlation coefficient (r) between variables and the dimensions (Dim) were considered significant with p-values <0.05. Nonmetric multidimensional scaling (NMDS) ordination compared bacterial population differences, using distance analysis in Phyloseq. A Phyloseq-generated heatmap visualized rodent blood OTU diversity and abundance. Permutational ANOVA (PerMANOVA), using vegan’s adonis function and Bray-Curtis index with 1000 permutations (Oksanen et al., 2016) tested habitat-based bacterial composition differences, with significance at pseudo F-associated p-values ≤0.05.

### 2.7 *Bartonella gltA* gene characterization

Characterization of *Bartonella* spp. in rodents was done using primers Bart-EF and Bart-ER targeting the citrate synthase (*gltA*) gene yielding approximately 500 bp fragments (Bastos, 2007). The PCR products were sequenced, and sequences submitted to Genbank (accession numbers: PP831190-PP831198). Sequence alignment was performed with closely related sequences, identified through nucleotide BLAST search. Phylogenetic analysis utilized the Maximum Likelihood method, applying the best-fit evolutionary model in MEGA 11 (Tamura et al., 2021).

## 3 Results

### 3.1 Molecular typing of *Mastomys* hosts

Analysis of the COI gene classified 10 out of 25 *Mastomys* specimens as *M. coucha*, one as *Steatomys* sp., and the remainder as *M. natalensis*. This was corroborated by *cytb* gene sequencing (**Supplementary Table S2**). Habitat distribution patterns indicated a prevalence of *M. natalensis* in urban and peri-urban settings, whereas *M. coucha* was more frequently encountered in communal rangelands and protected areas. Closer examination of the COI gene sequences from *M. natalensis* revealed minor variations, with individual specimens R31, R84, and R74 exhibiting single nucleotide differences at specific loci within a 650-nucleotide sequence. Greater genetic diversity was observed in *M. coucha*, with a 658-nucleotide sequence analysis showing single nucleotide variations among specimens R2, R12, R159, R177, and R179. Notably, R2 and R12 shared identical sequences, as did R159 and R177. Additionally, unique nucleotide substitutions were identified in specimens R5, R6, R11, R20, and R61, each at distinct positions.

### 3.2 Ectoparasites

A total of 439 ectoparasites comprising 10 ticks (4 *Haemaphysalis leachi/elliptica* group, 4 *Rhipicephalus simus/follis* group and 2 *Rhipicephalus microplus*), 337 mites of which 5 were trombiculid mites, 61 fleas (mainly *Xenopsylla brasilliensis* at >30% prevalence) and 31 lice (21 *Polyplax biseriata* and 10 *Holopleura intermedia*) were collected from the rodents (**Table 1**).

**Table 1:** Ectoparasites recovered from *Mastomys* spp. and *Steatomys*.

Rodent species	Sample number	Ticks						Mites	Fleas	Lice	TM^a^
		*H. leachi/elliptica*		*R. microplus*		*R. simus*/*follis*					
		larva	nymph	larva	nymph	larva	nymph				
*M. natalensis*	R19	–	–	–	–	–	–	8	2	–	–
*M. coucha*	R20	–	–	–	–	–	–	1	7	–	–
*M. natalensis*	R29	–	–	–	–	–	–	7	–	3	–
*M. natalensis*	R30	3	1	–	–	–	–	12	–	–	–
*M. natalensis*	R31	–	–	–	–	–	–	12	–	1	–
*M. natalensis*	R171	–	–	–	–	–	–	80	–	2	2
*M. natalensis*	R172	–	–	–	–	–	–	7	2	–	2
*M. natalensis*	R74	–	–	–	–	–	–	10	7	–	–
*M. natalensis*	R75	–	–	–	–	–	–	7	1	–	–
*M. natalensis*	R78	–	–	–	–	–	–	2	4	–	–
*M. natalensis*	R95	–	–	–	–	–	–	–	–	–	–
*M. natalensis*	R98	–	–	–	–	–	–	25	–	–	–
*M. natalensis*	R99	–	–	–	–	–	–	–	–	–	–
*M. coucha*	R2	–	–	–	–	–	–	2	4	2	–
*M. coucha*	R5	–	–	1	–	–	2	5	8	–	–
*M. coucha*	R6	–	–	1	–	–	2	19	8	–	–
*M. coucha*	R11	–	–	–	–	–	–	25	3	1	–
*M. coucha*	R12	–	–	–	–	–	–	9	6	1	–
*M. natalensis*	R84	–	–	–	–	–	–	24	1	–	–
*M. natalensis*	R21	–	–	–	–	–	–	7	1	–	–
*Steatomys* sp.	R53	–	–	–	–	–	–	14	–	–	–
*M. coucha*	R61	–	–	–	–	–	–	8	3	–	–
*M. coucha*	R159	–	–	–	–	–	–	13	4	–	–
*M. coucha*	R177	–	–	–	–	–	–	6	–	21^#^	1
*M. coucha*	R179	–	–	–	–	–	–	29	–	–	–

a Trombiculid mites; ^#^ *Polyplax biseriata*, the rest of the lice were *Hoplopleura intermedia*.

### 3.3 Barcoded 16S rRNA gene sequencing and statistical analysis

**Figure 1:**
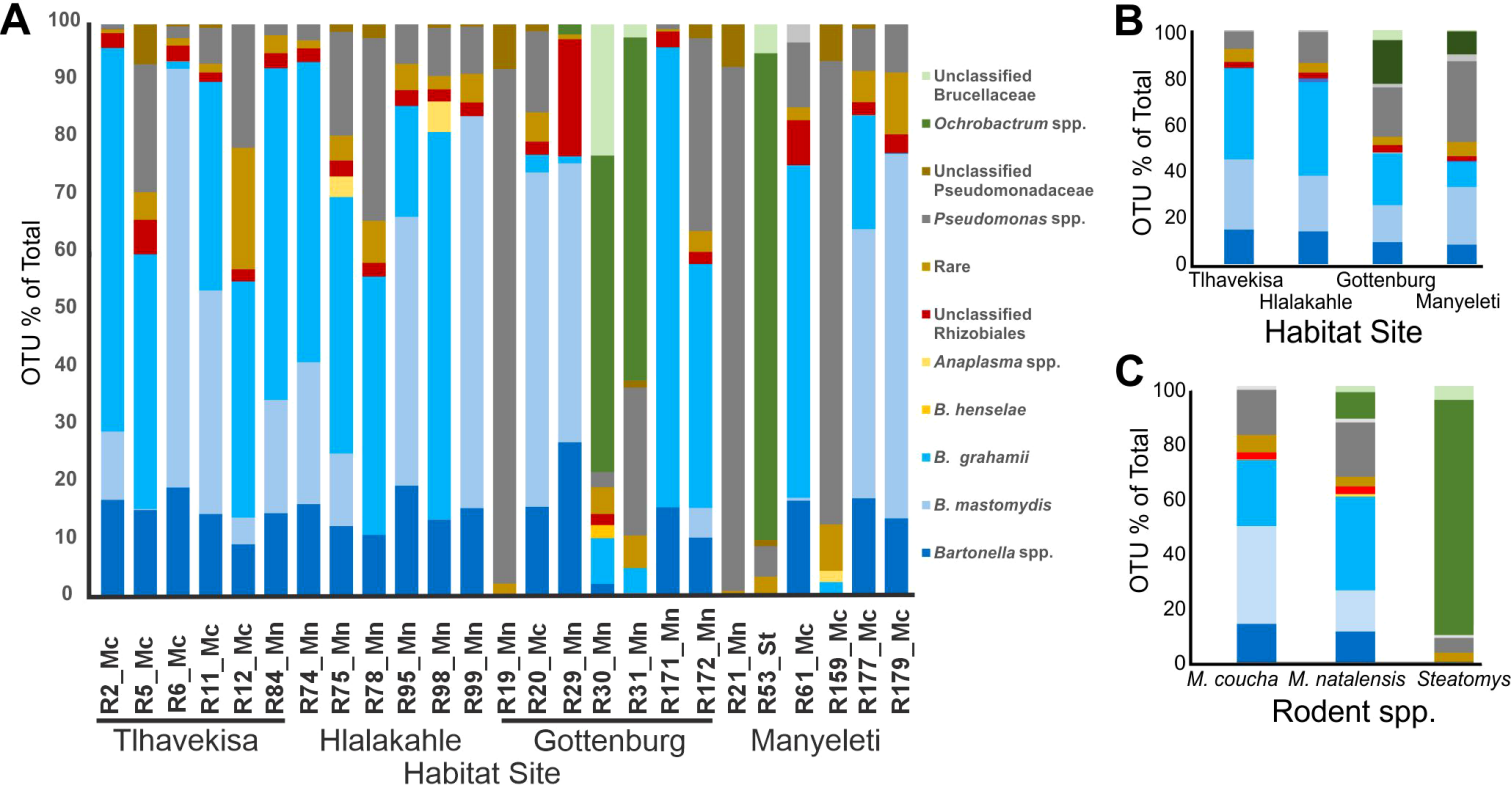
Relative abundance of major taxa of bacteria. **(A)** In individual rodents, six to seven rodents from each habitat area were sampled. Rodent species are linked to sample numbers (Mn abbreviation for *M. natalensis*, Mc for *M. coucha* and St for *Steatomys*). The village names are indicated under the rodent sample numbers. **(B)** In the blood of *Mastomys* spp. from different habitat areas: Hlalakahle and Gottenburg (urban/peri-urban area), Tlhavekisa (communal rangeland), and Manyeleti (protected wild reserve) and **(C)** In the blood of *Mastomys coucha, M. natalensis* and *Steatomys* sp.

Sequencing of the barcoded 16S amplicons generated 65,059 bacterial sequences, with a mean of 2,602 reads per sample, confirming the capture of all OTUs to satisfy a rarefaction curve (**Supplementary Figure S2**). The species diversity, represented by a plateau in the rarefaction curve, indicated a well-sampled bacterial community. Analysis identified 17 OTUs across ten species and six genera, excluding rare and unclassified OTUs. Approximately 90.0% of the total reads (58,543 out of 65,059) were assigned to valid taxa. *Bartonella grahamii* was the most prevalent, forming 29.0% of the bacterial sequences, followed by *Bartonella mastomydis* at 23.0%. *Bartonella* spp. that fell below the 98% cut-off point made up 12.0% of the sequences. Other bacteria included *Pseudomonas* spp. (17.9%), *Ochrobactrum* spp. (7.2%), and *Anaplasma* spp. (0.5%), *B. henselae* (0.1%), *Ehrlichia* sp. (0.03%), and *Coxiella burnetii* (0.02%). *Anaplasma* spp. comprised 10 sequences of *A. centrale*, five sequences of *A. phagocytophilum* and two sequences of *A. marginale*. Rare and unclassified OTUs constituted 4.7% and 5.3% of the sequences. Predominant bacterial taxa are illustrated in **Figure 1A**.

#### 3.3.1 OTUs by habitat area

A heat map of OTU diversity in rodent blood, grouped by capture location (**Supplementary Figure S3**) revealed high *Bartonella* spp. prevalence (~83%) in rodents from Hlalakahle (peri-urban) and Tlhavekisa (communal rangeland), contrasting with lower occurrences (~45%) in Gottenburg (peri-urban) and Manyeleti (wildlife reserve). *Ochrobactrum* spp. was detected in Gottenburg and Manyeleti specimens. *Ehrlichia* sp. was detected in R75 (Hlalakahle) and R172 (Gottenberg), while *Coxiella* sp. was exclusive to rodent R12 (Tlhavekisa). *Anaplasma phagocytophilum* was identified in Rodent R98 (Hlalakahle) while *A. centrale* and *A. marginale* were identified in R20 (Gottenburg). Manyeleti rodents exhibited the highest *Pseudomonas* spp. infection rates (~34%), influenced by significant burdens in rodents R21 and R159. The microbial profile of rodent blood, based on rodent capture sites, is depicted in **Figure 1B**.

#### 3.3.2 OTUs by rodent species

Based on rodent species, *B. grahamii* accounted for 23.9% and 33.9% of sequences in *M. coucha* and *M. natalensis*. *Bartonella mastomydis* was more prevalent in *M. coucha* at 35.4%, compared to 14.9% in *M. natalensis*. Unspeciated *Bartonella* were comparably lower, constituting 13.9% in *M. coucha* and 11.1% in *M. natalensis*. Notably, *B. henselae* was exclusively found in *M. natalensis*, albeit a minimal 0.2%. *Pseudomonas* spp. showed a higher presence in *M. natalensis* (19.7%) than in *M. coucha* (16.4%), and *Steatomys* (5.3%). Conversely, *Ochrobactrum* spp. dominated in *Steatomys* with 85.1% and was less common in *M. natalensis* at 9.6%. The microbial composition in the blood of *M. coucha*, *M. natalensis*, and *Steatomys* sp. is shown in **Figure 1C**.

#### 3.3.3 Relative abundance of taxa

Most *Bartonella* sequences had 99% identity to *B. grahamii* strain as4aup (CP001562), while a subset matched *B. mastomydis* (AY993936). *Ehrlichia* sequences in rodents R75 and R172 showed 98% identity to strains EH727 (AY309970) and Ehf669 (AY309969), as well as *Ehrlichia* sp. Tibet (AF414399) and *E. chaffeensis* (NR_074500). *Anaplasma* sequences in rodent R98 were 99% identical to several *A. phagocytophilum* strains, including the Dog2 strain (CP006618) and human strain HZ (CP000235). *Ochrobactrum* spp. detected in rodents from Gottenburg and Manyeleti, showed 99% identity to *O. intermedium* (JN613288 & KT696500), and *O. pseuintermedium* (DQ365922). *Pseudomonas* spp., with 98% identity to *P. extremaustralis* (NR114911), *Pseudomonas* sp. BFXJ-8 (EU013945) and *P. fluorescens* (CP015638), were found in all rodents except R29. Lastly, rodent R12 had sequences with 99% identity to *C. burnetii* strains Schperling (CP014563) and Namibia (CP007555).

#### 3.3.4 PCA and NMDS analyses

**Figure 2:**
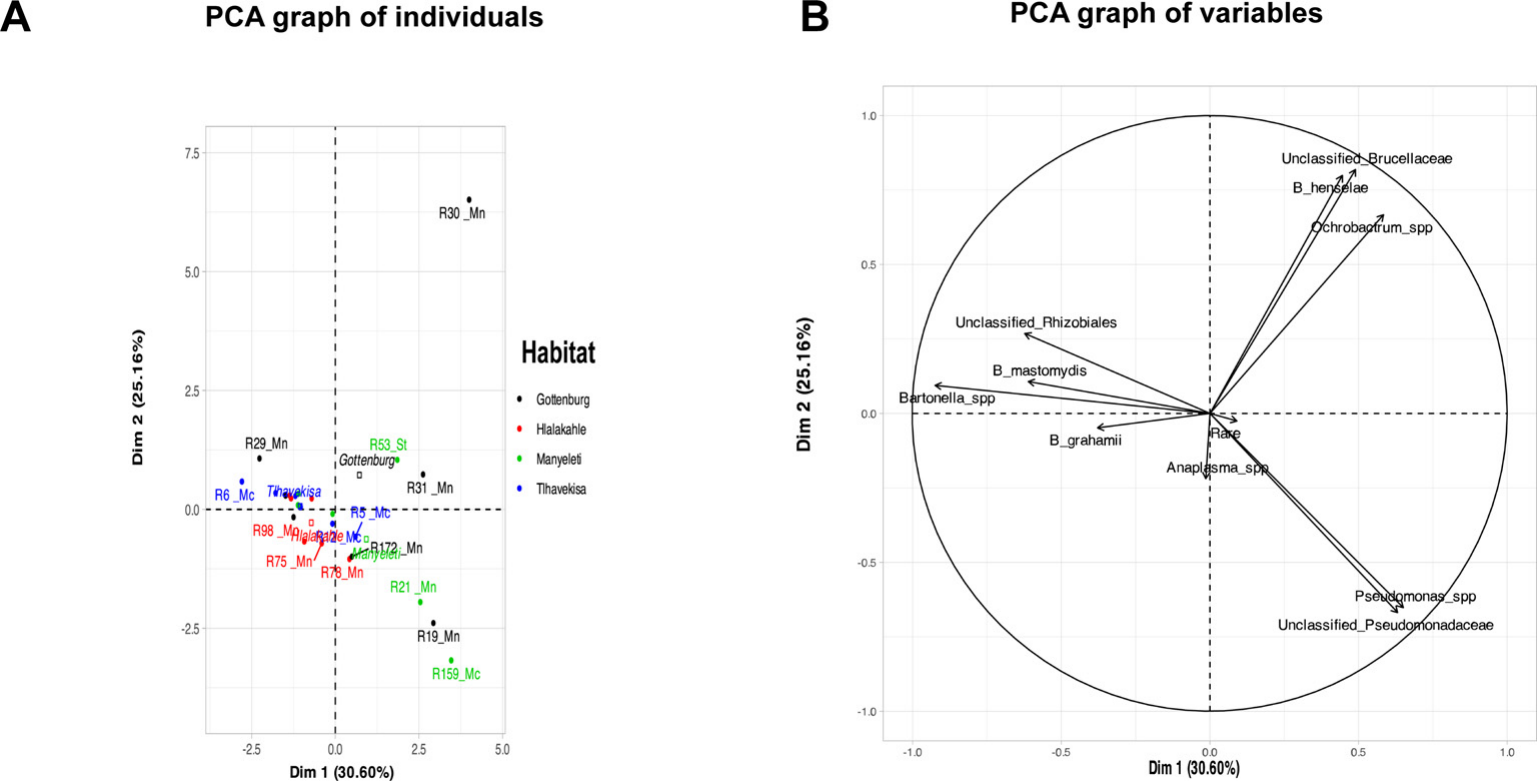
PCA plot analyzing the blood microbiome of rodents with the first two principal components explaining 30.60% and 25.16% of the variance, respectively. **(A)** Graph of individual rodents. Black dots represent samples from Gottenburg, red samples are from Hlalakahle, green samples are from Manyeleti and blue samples from Tlhavekisa. Clustering suggests similarities in bacterial profiles and dispersion indicates variability. Notably, R30 from Gottenburg is an outlier. Rodent species are linked to sample numbers (Mn abbreviation for *M. natalensis*, Mc for *M. coucha* and St for *Steatomys*). The position of habitat names reflects their significant contribution to the plot’s dimensions. **(B)** Graph of variables shows bacterial populations detected from rodent blood in the Bushbuckridge-East community.

PCA showed 30.60% of the variation in the rodent bacterial communities clustered with Dim1, while 25.16% clustered with Dim2. Rodent R30 from Gottenburg was distinct, while R19 (Gottenburg), R21 and R159 (Manyeleti) showed similarities, clustering at the mid to bottom right. Other rodents, R6, R29, R31, and R53, varied, while the rest formed a main cluster (**Figure 2A**). The variables factor map (**Figure 2B**) showed that *Pseudomonas* spp. (R= 0.7), unclassified Pseudomonadaceae (R= 0.6), *Ochrobactrum* spp. (R=0.6), unclassified *Brucellaceae* (R=0.5), and *B. henselae* (R= 0.4) were positively correlated with Dim 1. Conversely, *Bartonella* spp. (R= -0.9), *B. mastomydis* (R= -0.6), and unclassified Rhizobiales (R=0.6) correlated negatively with Dim 1 and positively with Dim 2. R30 and R31 (Gottenburg), and R53 (Manyeleti) were associated with variables positively affecting Dim 1, while the main cluster was linked to variables influencing Dim 2. Finally, R19 (Gottenburg), R21 and R159 (Manyeleti) correlated with *Pseudomonas* spp. and unclassified *Pseudomonadaceae*.

The NMDS plot displayed variations in bacterial diversity of rodent blood across habitats and among *M. coucha*, *M. natalensis*, and *Steatomys* species (**Supplementary Figure S3**). Despite this, PerMANOVA revealed no significant differences in bacterial populations between rodents from Tlhavekisa (rangelands) and Hlalakahle (urban/per-urban area) (P = 0.1). The study’s small sample size limited our ability to draw statistically significant conclusions regarding the diversity of the bacterial blood microbiome of the rodents across the habitats.

### 3.4 Phylogenetic analysis of *Bartonella* spp.

#### 3.4.1 16S rRNA gene phylogeny

**Figure 3:**
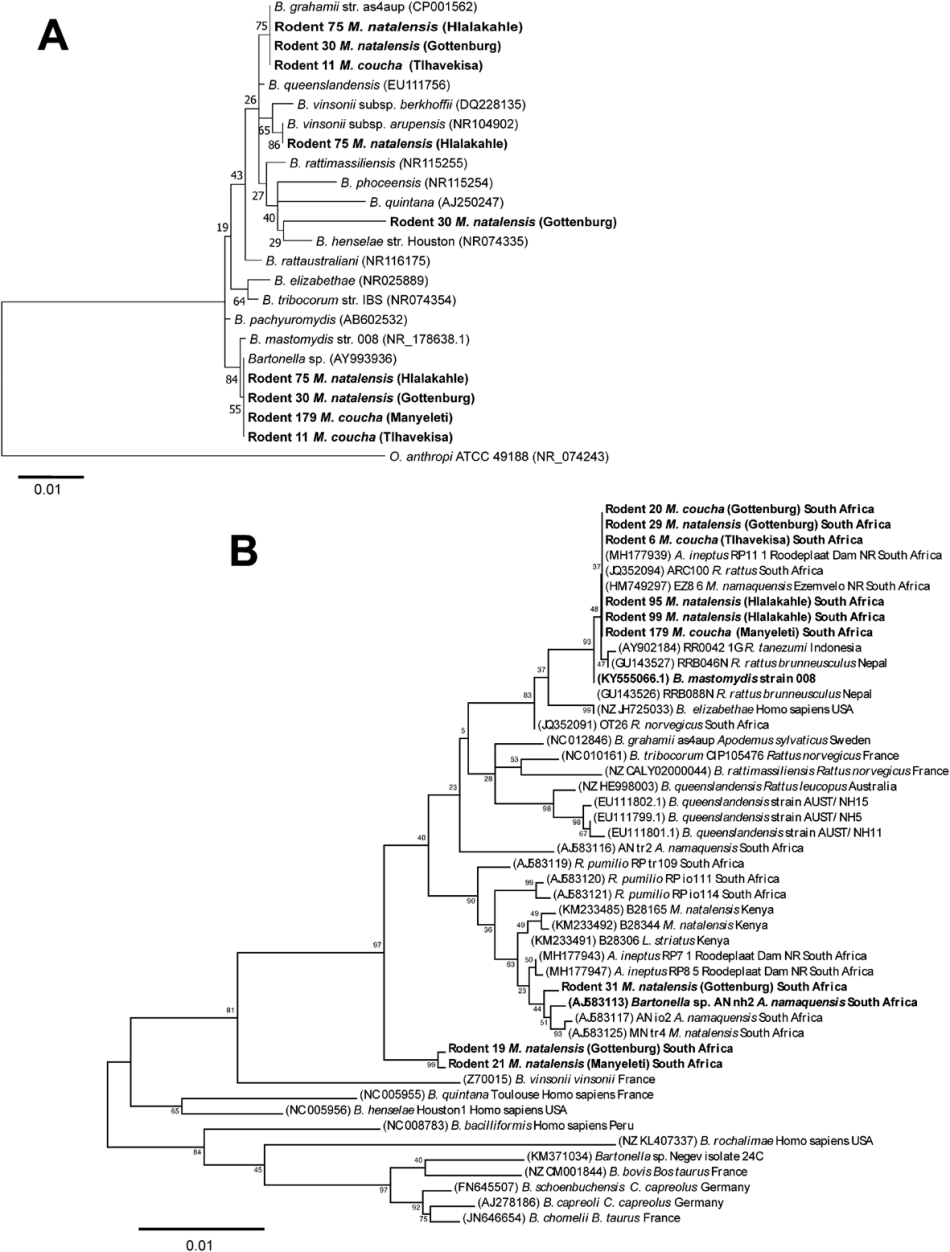
Maximum likelihood trees. **(A)** 16S rRNA gene tree inferred using the General Time Reversible (GTR) model of sequence evolution with G + I [G (0.1000), I (46.51% sites)]. Relationships between previously described *Bartonella* spp. and the *Bartonella* strains identified in the blood of five rodents from different habitat areas in the Bushbuckridge-East community (indicated in bold) are shown. Three rodents (11, 30 and 75) were co-infected with more than one *Bartonella* strains. Genbank accession numbers are given in parenthesis. The analysis involved 24 nucleotide sequences. There were 1309 positions in the final dataset. **(B)** *gltA* gene tree inferred using the Tamura 3-parameter + G (0.25) model of sequence evolution. *Bartonella* spp. identified in the blood of rodents from different habitat areas in the Bushbuckridge-East community and reference *Bartonella* sequences are indicated in bold, with rodents 6 and 179, common to both the 16S rRNA and *glt*A gene phylogenies. Genbank accession numbers are given in parenthesis. The analysis involved 47 nucleotide sequences. Bootstrap values from 1000 replications are indicated next to the relevant node. The tree is drawn to scale, with branch lengths measured in the number of substitutions per site.

Five rodents (R6, R11, R30, R75 and R179) from the different habitats had one or more *Bartonella* spp. co-infections. These sequences (8) were used for phylogenetic analysis of the 16S rRNA gene using the Maximum likelihood method (**Figure 3A**).

#### 3.4.2 *Glt*A gene phylogeny

Sequence analysis of *gltA* gene sequences from R5, R11, R12, and R84 captured from Tlhavekisa, identified multiple *Bartonella* co-infections complicating species delineation. However, nine rodents yielded clean/unambiguous nucleotide sequences. Notably, six samples (R6, R20, R29, R95, R99, R179) from the various habitats were infected with *B. mastomydis* (**Figure 3B**). Potentially novel *Bartonella* were identified in *M. natalensis*: one in R19 and R21 (from Gottenburg and Manyeleti) which formed a distinct clade separate from known *Bartonella* spp., and the second in R31 (Gottenburg), closely related to *Bartonella* sp. AN-nh2 previously detected in *Micaelamys namequensis* (Pretorius et al., 2004). Sequences from R6 and R179 contributed to both 16S rRNA and *gltA* gene phylogenies.

## 4 Discussion

This study identified a variety of bacterial species in the blood of *Mastomys* and *Steatomys* spp., predominantly *Bartonella* spp., at 64% of sequences. All rodents were infected, aligning with prior research that showed *Bartonella*’s prevalence in rodent blood and fleas in Israel and the US (Cohen et al., 2015; Rynkiewicz et al., 2015), although Rynkiewicz et al. (2015) reported a higher infection rate (97.8%). In this study, *B. grahamii* represented 33.9% and 23.9% of bacterial sequences in *M. natalensis* and *M. coucha*. Previously detected in *Gerbilliscus leucogaster* in South Africa and *Rattus norvegicus* in Nigeria (Pretorius et al., 2004; Kamani et al., 2013), *B. grahamii* is associated with retinal occlusions, cat scratch disease and neuroretinitis in humans (Kerkhoff et al., 1999; Serratrice et al., 2003; Oksi et al., 2013). The co-infection with other *Bartonella* spp. highlights the need for further studies on *Bartonella’*s pathogenicity and transmission dynamics, given its public health implications. The presence of zoonotic *B. grahamii* in local rodents underscores the potential health risks to the community.

Rodents in peri-urban and communal rangelands had higher *Bartonella* burdens compared to those in protected areas, indicating a correlation between *Bartonella* spp. prevalence and human proximity.

*Bartonella mastomydis* constituted a significant portion of bacterial sequences in *M. coucha* (35.4%) and *M. natalensis* (14.9%). The organism has previously been detected in *R. tanezumi flavipectus* in China (Li et al., 2004). Its presence in South African rodents coupled with the molecular confirmation of invasive *R. tanezumi* in South Africa (Bastos et al., 2011) suggests spillover of infection between invasive and indigenous species (Hatyoka et al., 2019a).

Analysis of *Bartonella gltA* gene sequences confirmed *B. mastomydis* in *M. natalensis* and *M. coucha* across habitats. This strain has been found in other South African murids including *Aethomys ineptus*, *Micaelamys natalensis* and *R. rattus* (Mostert, 2010; Brettschneider et al., 2012; Hatyoka et al., 2019a). These studies pre-date the formal description of this species, all noting the close relationship of these strains to *Bartonella elizabethae*, a zoonotic species, thus 16S rRNA sequencing may misclassify *B. mastomydis* as *B. elizabethae* (Kosoy et al., 2018). These results represent the first confirmation of this organism in *Mastomys* in South Africa. Phylogeny of the *gltA* gene revealed a new *Bartonella* sp. in two *M. natalensis* specimens from Gottenburg and Manyeleti. Another taxon closely related to a *Bartonella* sp. AN-nh2 previously detected in *Micaelamys namaquensis* from the Free state, South Africa (Pretorius et al., 2004) was detected in R31 (*M. natalensis*) from Gottenburg.

*Bartonella henselae*, known for causing endocarditis and occult infections in immunocompromised humans (Hadfield et al., 1993; Breitschwerdt et al., 2007), was detected in R30 (Gottenburg). *Bartonella henselae* has been previously detected in cats and humans in South Africa (Kelly, 1996; Frean et al., 2002; Trataris et al., 2012). Its prior detection in two AFI patients in Bushbuckridge-East (Simpson et al., 2018) and now in *Mastomys* spp. from the same area indicates its potential role in human infections.

Our finding that *Mastomys* spp. across habitat areas were concurrently infected with multiple *Bartonella* spp. aligns with prior research showing a variety of *Bartonella* spp. in Chinese and Spanish rodents (Ying et al., 2002) (Márquez et al., 2008). Similarly, three distinct *Bartonella* lineages were detected in *Rhabdomys pumilio* (Western Cape) and *A. ineptus* (Gauteng) (Hatyoka et al., 2019a, 2019).

As arthropod vectors transmit *Bartonella* spp. (Breitschwerdt and Kordick, 2000), we examined the ectoparasites from the rodents, recovering 439 ectoparasites. Regulations of the South African Department of Agriculture, Land Reform and Rural Development (DALRRD) [which stated that whole body rodent carcasses must first be frozen at -80°C and stored at Hans Hoheisen Research Centre, Mpumalanga South Africa before transport on dry ice to a BSL3 provincial laboratory at Stellenbosch, South Africa where ectoparasites could be removed] prevented immediate screening of the ectoparasites for *Bartonella* spp. or any other pathogens at the time of the rodent capture. Nevertheless, the findings of fleas, ticks and mites is consistent with vectored transmission. Results of ectoparasite screening studies are currently ongoing and will be reported at a later time.

Overall, 17% of bacterial sequences in rodent blood corresponded to *Pseudomonas* spp., primarily from R21 and R159, from Manyeleti, with the organism making 91.6% and 81% of the total sequences obtained from the two samples. *Pseudomonas* spp. are opportunistic pathogens causing infection in immunocompromised rodents (Baker, 1998), yet their presence could stem from contamination, as they are known contaminants in 16S rRNA gene sequencing (Salter et al., 2014). DNA extraction kits and other laboratory reagents have also been implicated as sources of bacterial DNA contamination in microbiome studies (Salter et al., 2014). However, physical signs like tail growths and foot deformities associated with bumblefoot (ulcerative pododermatitis) observed in some of rodents in this study suggest valid *Pseudomonas* infections (Blair, 2013) (**Supplementary Figure S5**).

*Ochrobactrum* spp. made up 7.2% of sequences from rodent blood, in *M. natalensis* (R29, R30, R31) from Gottenburg and *Steatomys* (R53) from Manyeleti. In R53, *Ochrobactrum* dominated comprising 85% of the total sequences. Recognized as opportunistic nosocomial pathogens (Hagiya et al., 2013), *Ochrobactrum* can present clinical symptoms that mimic the more virulent *Brucella* spp. Their close phylogenetic relationship has led to discussions on unifying *Ochrobactrum* and *Brucella*, with dissenting views (Hördt et al., 2020; Oren and Garrity, 2020). The CDC has recently unified these taxa (CDC, 2022) and the American Society for Microbiology Clinical and Public Health Microbiology Committee suggested that clinical laboratories report the affected organisms as *Brucella* (*Ochrobactrum*) species to differentiate them from the archetypal *Brucella* agent (She et al., 2023). Recently, 78 scientists highlighted significant flaws in the proposed nomenclature, citing insufficient phylogenetic analysis and exclusion of expert opinion, which could lead to serious risks for personnel handling zoonotic *Brucella*, particularly in poorer nations (Moreno et al., 2023). Therefore, based on the current knowledge on both organisms, we agree that *Ochrobactrum* and *Brucella* spp. be maintained as separate genera and have treated them as such.

*Anaplasma centrale* and *A. marginale* were detected in R20 (*M. coucha*) from Gottenburg and *A. phagocytophilum* in R98 (*M. natalensis*) from Hlalakahle. Typically, *A. marginale* and *A. centrale* infect cattle and other wild ruminant species (Aubry and Geale, 2011; Hosseini-Vasoukolaei et al., 2014; Wu et al., 2015; Khumalo et al., 2016, 2018). The detection of *A. marginale* and *A. centrale* in wild rodents was surprising as these species are thought to only infect ruminants. Because the number of sequences obtained was low, and sequence coverage was high (Rhoads and Au, 2015), their detection could be due to salivary secretions from infected ticks rather than successful establishment of infection. However, rodents in this study had a low tick burden and the *Anaplasma* positive rodents had no ticks on them at the time of capture. *Anaplasma phagocytophilum* detection in rodents, dogs and cattle from the study area has been previously reported (Kolo et al., 2020).

An *Ehrlichia* sp. was identified in R75 from Hlalakahle and R172 from Gottenburg (*M. natalensis*) with 99% identity to *Ehrlichia* sp. Ehf669 (AY309969) detected from *Haemaphysalis* ticks collected from dogs in Japan (Inokuma et al., 2004) and 98% identity to *E. chaffeensis* (NR_074500). *Ehrlichia chaffeensis* causes human monocytic ehrlichiosis (HME), a tick-borne zoonosis in the US transmitted by *Amblyomma americanum* ticks (Paddock and Childs, 2003). Previously, antibodies against *E. chaffeensis* have been detected in dogs in the Free State (Pretorius and Kelly, 1998). This study reports the first finding of an *E. chaffeensis*-like sequence in South African rodents.

*Coxiella burnetii*, the causative agent of Q fever, was detected in R12 (*M. coucha*) from Tlhavekisa marking its first detection from a wild rodent in South Africa. Q fever, manifesting as an acute febrile illness and chronic endocarditis in humans, and linked to livestock abortion (Vanderburg et al., 2014), is transmitted by ticks, aerosols or consumption of contaminated animal products (Maurin and Raoult, 1999). In South Africa, *C. burnetii* antibodies have been detected in cattle (Gummow et al., 1987), and wild dogs in the Kruger Park (Van Heerden et al., 1995).

We did not detect any *Rickettsia* spp. in rodent blood in this study contrasting with Essbauer et al. (2018) who detected pathogenic *R. conorii*, *R. massiliae*, *R. felis* and *R. helvetica*, in ear tissue of rodents sampled across South Africa and Namibia. Rickettsial pathogens are usually found in the dermis, vascular endothelium and spleen (Hawley et al., 2007; Bayliss et al., 2009) which may explain the absence of detection in blood samples. Essbauer et al. (2018) did not report any *Rickettsia* in *M. natalensis*, but found a 9% infection rate in *M. coucha*, which were identified as “*Candidatus* Rickettsia africaustralis”.

The detection of *Anaplasma*, *Ehrlichia*, *B. henselae*, and *Coxiella* in *Mastomys* spp. in this study was minimal, possibly lacking statistical significance. However, their zoonotic and veterinary significance warranted emphasis.

PCA analysis showed that rodents from Tlhavekisa and Hlalakahle shared similar blood microbiome profiles as opposed to rodents from Tlhavekisa and Gottenburg rodents which had distinct profiles. This is despite Gottenburg being closer to Tlhavekisa than Hlalakahle (7.7 km vs 8.1 km). The blood microbiome of Manyeleti rodents was also distinct, with *Pseudomonas* and *Ochrobactrum* spp. dominance. This finding supports studies by Gavish et al. (2014) and Gall et al. (2017) that suggested factors like geography, host diversity, and human interaction might influence bacterial diversity in hosts and vectors. PCA also revealed an association of positive correlations between *Bartonella* spp. and unclassified Rhizobiales and between *Ochrobactrum* spp., unclassified Brucellaceae, and *B. henselae*. Overall, the small sample size meant individual variations significantly influenced the results.

Our results suggest that the rodent blood microbiome is relatively species-sparse. Although the mean sequencing depth was low, the rarefaction curves suggest that the species richness in all samples was adequately captured, and further sequencing would not significantly increase the number of observed species. It is possible that the observed pattern reflects some degree of bias towards dominant species as PCR-based amplification and sequencing methods often favor highly abundant sequences, which may overshadow less abundant or rare species. Under-sampling rare species is a known limitation of low sequencing depths, and future studies could address this by increasing the sequencing depth to capture rare OTUs.

The study identified 17 OTUs across ten species and six genera, with *Bartonella grahamii* and *B. mastomydis* dominating the dataset. Approximately 90.0% of the total reads could be assigned to valid taxa, while reads classified as “Rare” accounted for 4.7% of the total reads. This taxonomic distribution indicates that the majority of the reads were assigned to non-rare taxa, suggesting a skew toward dominant species in the microbiome. These findings correspond with previous studies that show rodent blood microbiomes are often dominated by a few bacterial species due to selective pressures and niche specificity in the bloodstream environment (Gavish et al., 2014; Rynkiewicz et al., 2015). Another study found that the rodent blood microbiome is influenced by factors such as host immunity and interspecific bacterial interactions that favors the dominance of specific bacterial species (Cohen et al., 2015). This species-sparse nature of the rodent blood microbiome may explain why a mean sequencing depth of 2,602 reads was sufficient to capture the majority of OTUs present.

In conclusion, this study provides foundational data on bacterial diversity in the blood of indigenous murid rodents and highlights *Mastomys* spp. as key reservoirs of bartonellae. It reports the first confirmation of *B. mastomydis* detection in two cryptic *Mastomys* species in South Africa and documents the detection of important zoonotic pathogens including *Ehrlichia* spp., and *C. burnetii.*

## Data Availability

The datasets presented in this study can be found in online repositories. The names of the repository/repositories and accession number(s) can be found in the article/**Supplementary Material**.

## References

[B1] Aubry P. Geale D. (2011). A review of bovine anaplasmosis. Transbound Emerg. Dis. 58, 1–30. doi: 10.1111/j.1865-1682.2010.01173.x 21040509

[B2] Baker D. G. (1998). Natural pathogens of laboratory mice, rats, and rabbits and their effects on research. Clin. Microbiol. Rev. 11, 231–266. doi: 10.1128/CMR.11.2.231 9564563 PMC106832

[B3] Bastos A. D. (2007). *Bartonella* incidence and diversity in endemic South African murid rodents occurring commensally with humans. S. Afri. Soc Vet. Epidemiol. Prev. Med., 78–83.

[B4] Bastos A. D. Chimimba C. T. Von Maltitz E. Kirsten F. Belmain S. R. (2005). Identification of rodent species that play a role in disease transmission to humans in South Africa. Proc. S. Afr. Soc Vet. Epidemiol. Prev. Med., 78–83. doi: 10.13140/2.1.2629.4563

[B5] Bastos A. D. Nair D. Taylor P. J. Brettschneider H. Kirsten F. Mostert E.. (2011). Genetic monitoring detects an overlooked cryptic species and reveals the diversity and distribution of three invasive *Rattus* congeners in South Africa. BMC Genet. 12, 26–26. doi: 10.1186/1471-2156-12-26 21324204 PMC3055845

[B6] Bayliss D. B. Morris A. K. Horta M. C. Labruna M. B. Radecki S. V. Hawley J. R.. (2009). Prevalence of *Rickettsia* species antibodies and *Rickettsia* species DNA in the blood of cats with and without fever. J. Feline Med. Surg. 11, 266–270. doi: 10.1016/j.jfms.2008.06.007 18786845 PMC10911462

[B7] Berrian A. M. van Rooyen J. Martínez-López B. Knobel D. Simpson G. J. G. Wilkes M. S.. (2016). One Health profile of a community at the wildlife-domestic animal interface, Mpumalanga, South Africa. Prev. Vet. Med. 130, 119–128. doi: 10.1016/j.prevetmed.2016.06.007 27435655

[B8] Blair J. (2013). Bumblefoot: a comparison of clinical presentation and treatment of pododermatitis in rabbits, rodents, and birds. Vet. Clin. North Am. Exot. Anim. Pract. 16, 715–735. doi: 10.1016/j.cvex.2013.05.002 24018034

[B9] Bonnet S. Michelet L. Moutailler S. Cheval J. Hebert C. Vayssier-Taussat M.. (2014). Identification of parasitic communities within European ticks Using next-generation sequencing. PloS Negl. Trop. Dis. 8, e2753. doi: 10.1371/journal.pntd.0002753 24675738 PMC3967966

[B10] Bonwitt J. Sáez A. M. Lamin J. Ansumana R. Dawson M. Buanie J.. (2017). At home with *Mastomys* and *Rattus*: human-rodent interactions and potential for primary transmission of Lassa Virus in domestic spaces. Am. J. Trop. Med. Hyg. 96, 935–943. doi: 10.4269/ajtmh.16-0675 28167603 PMC5392645

[B11] Breitschwerdt E. B. Kordick D. L. (2000). *Bartonella* infection in animals: carriership, reservoir potential, pathogenicity, and zoonotic potential for human infection. Clin. Microbiol. Rev. 13, 428–438. doi: 10.1128/CMR.13.3.428 10885985 PMC88941

[B12] Breitschwerdt E. B. Maggi R. G. Duncan A. W. Nicholson W. L. Hegarty B. C. Woods C. W. (2007). *Bartonella* species in blood of immunocompetent persons with animal and arthropod contact. Emerg. Infect. Dis. 13, 938–941. doi: 10.3201/eid1306.061337 17553243 PMC2792845

[B13] Brettschneider H. Bennett N. C. Chimimba C. T. Bastos A. D. (2012). Bartonellae of the Namaqua rock mouse, *Micaelamys namaquensis* (Rodentia: Muridae) from South Africa. Vet. Microbiol. 157, 132–136. doi: 10.1016/j.vetmic.2011.12.006 22204791

[B14] Budachetri K. Browning R. E. Adamson S. W. Dowd S. E. Chao C. C. Ching W. M.. (2014). An insight into the microbiome of the *Amblyomma maculatum* (Acari: Ixodidae). J. Med. Entomol. 51, 119–129. doi: 10.1603/ME12223 24605461 PMC3956751

[B15] Centers for Disease Prevention and Control (2022). “12/19/2022: Lab Update: Reclassification of *Ochrobactrum* species into the *Brucella* genus” (C.f.D.C.a. Prevention).

[B16] Cohen C. Toh E. Munro D. Dong Q. Hawlena H. (2015). Similarities and seasonal variations in bacterial communities from the blood of rodents and from their flea vectors. ISME J. 9, 1662–1676. doi: 10.1038/ismej.2014.255 25575310 PMC4478706

[B17] Cole J. R. Wang Q. Cardenas E. Fish J. Chai B. Farris R. J.. (2009). The Ribosomal Database Project: improved alignments and new tools for rRNA analysis. Nucleic Acids Res. 37, D141–D145. doi: 10.1093/nar/gkn879 19004872 PMC2686447

[B18] Darriba D. Taboada G. L. Doallo R. Posada D. (2012). jModelTest 2: more models, new heuristics and parallel computing. Nat. Methods 9, 772. doi: 10.1038/nmeth.2109 PMC4594756 22847109

[B19] Davis D. H. (1964). Ecology of wild rodent plague. Reprinted from ecological studies in southern africa. Monogr. Biol. 14, 301–314. doi: 10.5555/19662901642

[B20] De Graaff G. (1981). The rodents of southern Africa: notes on their identification, distribution, ecology and taxonomy (Pretoria: Butterworths), 267.

[B21] Essbauer S. Hofmann M. Kleinemeier C. Wolfel S. Matthee S. (2018). *Rickettsia* diversity in southern Africa: A small mammal perspective. Ticks Tick Borne Dis. 9, 288–301. doi: 10.1016/j.ttbdis.2017.11.002 29174365

[B22] Frean J. Arndt S. Spencer D. (2002). High rate of *Bartonella henselae* infection in HIV-positive outpatients in Johannesburg, South Africa. Trans. R. Soc Tropi. Med. Hyg. 96, 549–550. doi: 10.1016/S0035-9203(02)90437-2 12474487

[B23] Gall C. A. Reif K. E. Scoles G. A. Mason K. L. Mousel M. Noh S. M.. (2016). The bacterial microbiome of *Dermacentor andersoni* ticks influences pathogen susceptibility. ISME J. 10, 1846–1855. doi: 10.1038/ismej.2015.266 26882265 PMC5029153

[B24] Gall C. A. Scoles G. A. Magori K. Mason K. L. Brayton K. A. (2017). Laboratory colonization stabilizes the naturally dynamic microbiome composition of field collected *Dermacentor andersoni* ticks. Microbiome 5, 133. doi: 10.1186/s40168-017-0352-9 28978338 PMC5628422

[B25] Gavish Y. Kedem H. Messika I. Cohen C. Toh E. Munro D.. (2014). Association of host and microbial species diversity across spatial scales in desert rodent communities. PloS One 9, e109677. doi: 10.1371/journal.pone.0109677 25343259 PMC4208758

[B26] Ge Y. Guo G. Ge B. Yin H. Yin H. (2018). The spleen microbiota of small wild mammals reveals distinct patterns with tick-borne bacteria. PloS Negl. Trop. Dis. 12, e0006499. doi: 10.1371/journal.pntd.0006499 29975692 PMC6033388

[B27] Greay T. L. Gofton A. W. Paparini A. Ryan U. M. Oskam C. L. Irwin P. J. (2018). Recent insights into the tick microbiome gained through next-generation sequencing. Parasitol. Vectors 11, 12. doi: 10.1186/s13071-017-2550-5 PMC5755153 29301588

[B28] Gummow B. Poerstamper N. Herr S. (1987). The incidence of *Coxiella burnetii* antibodies in cattle in the Transvaal. Onderstepoort J. Vet. Res. 54, 569–571. Available online at: https://pubmed.ncbi.nlm.nih.gov/3444611/ 3444611

[B29] Hadfield T. L. Warren R. Kass M. Brun E. Levy C. (1993). Endocarditis caused by *Rochalimaea henselae* . Hum. Pathol. 24, 1140–1141. doi: 10.1016/0046-8177(93)90196-N 8406424

[B30] Hagiya H. Ohnishi K. Maki M. Watanabe N. Murase T. (2013). Clinical characteristics of *Ochrobactrum anthropi* bacteremia. J. Clin. Microbiol. 51, 1330–1333. doi: 10.1128/JCM.03238-12 23363833 PMC3666774

[B31] Hatyoka L. M. Brettschneider H. Bennett N. C. Kleynhans D. J. Muteka S. P. Bastos A. D. S. (2019a). *Bartonella* diversity and zoonotic potential in indigenous Tete Veld rats (*Aethomys ineptus*) from South Africa. Infect. Genet. Evol. 73, 44–48. doi: 10.1016/j.meegid.2019.04.012 31004764

[B32] Hatyoka L. Froeschke G. Kleynhans D. van der Mescht L. Heighton S. Matthee S.. (2019b). Bartonellae of synanthropic four-striped mice (*Rhabdomys pumilio*) from the Western Cape Province, South Africa. Vector Borne Zoonotic Dis. 19, 242–248. doi: 10.1089/vbz.2018.2313 30571537

[B33] Hawley J. R. Shaw S. E. Lappin M. R. (2007). Prevalence of *Rickettsia felis* DNA in the blood of cats and their fleas in the United States. J. Feline Med. Surg. 9, 258–262. doi: 10.1016/j.jfms.2006.12.005 17276123 PMC10822615

[B34] Horak I. G. Heyne H. Williams R. Gallivan G. J. Spickett A. M. Bezuidenhout J. D.. (2018). The Ixodid Ticks (Acari: Ixodidae) of Southern Africa (Scotland, UK: Springer).

[B35] Hördt A. López M. G. Meier-Kolthoff J. P. Schleuning M. Weinhold L.-M. Tindall B. J.. (2020). Analysis of 1,000+ Type-strain genomes substantially improves taxonomic classification of Alphaproteobacteria. Front. Microbiol. 11. doi: 10.3389/fmicb.2020.00468 PMC7179689 32373076

[B36] Hosseini-Vasoukolaei N. Oshaghi M. A. Shayan P. Vatandoost H. Babamahmoudi F. Yaghoobi-Ershadi M. R.. (2014). *Anaplasma* infection in ticks, livestock and human in Ghaemshahr, Mazandaran Province, Iran. J. Arthropod. Borne Dis. 8, 204–211. Available online at: https://jad.tums.ac.ir/index.php/jad/article/view/210 26114134 PMC4478432

[B37] Huchon D. Madsen O. Sibbald M. J. Ament K. Stanhope M. J. Catzeflis F.. (2002). Rodent phylogeny and a timescale for the evolution of Glires: evidence from an extensive taxon sampling using three nuclear genes. Mol. Biol. Evol. 19, 1053–1065. doi: 10.1093/oxfordjournals.molbev.a004164 12082125

[B38] Inokuma H. Beppu T. Okuda M. Shimada Y. Sakata Y. (2004). Detection of ehrlichial DNA in *Haemaphysalis* ticks recovered from dogs in Japan that is closely related to a novel *Ehrlichia* sp. found in cattle ticks from Tibet, Thailand, and Africa. J. Clin. Microbiol. 42, 1353–1355. doi: 10.1128/JCM.42.3.1353-1355.2004 15004117 PMC356832

[B39] Jones R. T. Knight R. Martin A. P. (2010). Bacterial communities of disease vectors sampled across time, space, and species. ISME J. 4, 223–231. doi: 10.1038/ismej.2009.111 19865184

[B40] Kamani J. Morick D. Mumcuoglu K. Y. Harrus S. (2013). Prevalence and diversity of *Bartonella* species in commensal rodents and ectoparasites from Nigeria, West Africa. PloS Negl. Trop. Dis. 7, e2246. doi: 10.1371/journal.pntd.0002246 23738028 PMC3667778

[B41] Kelly D. (1996). *Bartonella* (Rochalimaea) *henselae* in southern Africa-evidence for infections in domestic cats and implications for veterinarians. J. S. Afr. Vet. Assoc. 67, 182–187. Available at: https://pubmed.ncbi.nlm.nih.gov/9284029/ 9284029

[B42] Kerkhoff F. T. Bergmans A. M. C. van der Zee A. Rothova A. (1999). Demonstration of *Bartonella grahamii* DNA in ocular fluids of a patient with neuroretinitis. J. Clin. Microbiol. 37, 4034–4038. doi: 10.1128/JCM.37.12.4034-4038.1999 10565926 PMC85873

[B43] Khumalo Z. T. H. Brayton K. A. Collins N. E. Chaisi M. E. Quan M. Oosthuizen M. C. (2018). Evidence confirming the phylogenetic position of *Anaplasma centrale* (ex Theiler 1911) Ristic and Kreier 1984. Int. J. Syst. Evol. Microbiol. 68, 2682–2691. doi: 10.1099/ijsem.0.002832 29916800

[B44] Khumalo Z. T. Catanese H. N. Liesching N. Hove P. Collins N. E. Chaisi M. E.. (2016). Characterization of *Anaplasma marginale* subspecies *centrale* strains using msp1aS genotyping reveals a wildlife reservoir. J. Clin. Microbiol. JCM, 54 (10), 01029–01016. doi: 10.1128/jcm.01029-16 PMC5035423 27440819

[B45] Kolo A. O. Collins N. E. Brayton K. A. Chaisi M. Blumberg L. Frean J.. (2020). *Anaplasma phagocytophilum* and other Anaplasma spp. in various hosts in the Mnisi Community, Mpumalanga Province, South Africa. Microorganisms 8, 1812. doi: 10.3390/microorganisms8111812 33217891 PMC7698776

[B46] Kosoy M. McKee C. Albayrak L. Fofanov Y. Genotyping of Bartonella bacteria and their animal hosts: current status and perspectives. Parasitology. (2018) 145 (5), 543–562. doi: 10.1017/S0031182017001263 28764816

[B47] Lê S. Josse J. Husson F. (2008). FactoMineR: an R package for multivariate analysis. J. Stat. Software 25, 1–18. doi: 10.18637/jss.v025.i01

[B48] Ledger J. (1980). The arthropod parasites of vertebrates in Africa south of the Sahara. Volume IV. Phthiraptera (Insecta) (Johannesburg: Johannesburg, South African Institute for Medical Research).

[B49] Li D. Yu D. Liu Q. Gong Z. (2004). Study on the prevalence of *Bartonella* species in rodent hosts from different environmental areas in Yunnan. Zhonghua Liu Xing Bing Xue Za Zhi 25, 934–937. Available online at: https://rs.yiigle.com/cmaid/591445 15769319

[B50] Luis A. D. Hayman D. T. S. O’Shea T. J. Cryan P. M. Gilbert A. T. Pulliam J. R. C.. (2013). A comparison of bats and rodents as reservoirs of zoonotic viruses: are bats special? Proc. R. Soc B: Biol. Sci. 280, 20122753. doi: 10.1098/rspb.2012.2753 PMC3574368 23378666

[B51] Márquez F. J. Rodríguez-Liébana J. J. Pachón-Ibáñez M. E. Docobo-Pérez F. Hidalgo-Fontiveros A. Bernabeu-Wittel M.. (2008). Molecular screening of *Bartonella* species in rodents from South Western Spain. Vector Borne Zoonotic Dis. 8, 695–700. doi: 10.1089/vbz.2007.0257 18620508

[B52] Maurin M. Raoult D. (1999). Q fever. Clin. Microbiol. Rev. 12, 518–553. doi: 10.1128/CMR.12.4.518 10515901 PMC88923

[B53] Moreno E. Middlebrook E. A. Altamirano-Silva P. Dahouk S. A. Araj G. F. Arce-Gorvel V.. (2023). If you’re not confused, you’re not paying attention: *Ochrobactrum* is not *Brucella* . J. Clin. Microbiol. 61, e00438–e00423. doi: 10.1128/jcm.00438-23 37395662 PMC10446859

[B54] Mostert M. (2010). Molecular and morphological assessment of invasive, inland rattus (Rodentia: Muridae) congenerics in South Africa and their reservoir host potential with respect to Helicobacter and Bartonella (Dissertation University of Pretoria (South Africa). Available online at: http://hdl.handle.net/2263/29390

[B55] Oksanen J. Blanchet F. Kindt R. Legendre P. O’Hara R. (2016). “Vegan: community ecology package. R Packag. 2.3-3”.). Available online at: http://vegan.r-forge.r-project.org/.

[B56] Oksi J. Rantala S. Kilpinen S. Silvennoinen R. Vornanen M. Veikkolainen V.. (2013). Cat scratch disease caused by *Bartonella grahamii* in an immunocompromised patient. J. Clin. Microbiol. 51, 2781–2784. doi: 10.1128/JCM.00910-13 23740723 PMC3719609

[B57] Oren A. Garrity G. (2020). List of new names and new combinations previously effectively, but not validly, published. Int. J. Syst. Evol. Microbiol. 70, 4043–4049. doi: 10.1099/ijsem.0.004244 32731908

[B58] Paddock C. D. Childs J. E. (2003). *Ehrlichia chaffeensis*: a prototypical emerging pathogen. Clin. Microbiol. Rev. 16, 37–64. doi: 10.1128/CMR.16.1.37-64.2003 12525424 PMC145301

[B59] Pretorius A. M. Beati L. Birtles R. J. (2004). Diversity of bartonellae associated with small mammals inhabiting Free State province, South Africa. Int. J. Syst. Evol. Microbiol. 54, 1959–1967. doi: 10.1099/ijs.0.03033-0 15545418

[B60] Pretorius A. Kelly P. (1998). Serological survey for antibodies reactive with *Ehrlichia canis* and *E. chaffeensis* in dogs from the Bloemfontein area, South Africa. J. S. Afr. Vet. Assoc. 69, 126–128. doi: 10.4102/jsava.v69i4.840 10192085

[B61] Razzauti M. Galan M. Bernard M. Maman S. Klopp C. Charbonnel N.. (2015). A comparison between transcriptome sequencing and 16S metagenomics for detection of bacterial pathogens in wildlife. PloS Negl. Trop. Dis. 9, e0003929. doi: 10.1371/journal.pntd.0003929 26284930 PMC4540314

[B62] R Core Team (2013). R: A language and environment for statistical computing (Vienna, Austria: R Foundation for Statistical Computing). Available at: http://www.R-project.org/.

[B63] Rhoads A. Au K. F. (2015). PacBio sequencing and its applications. Genomics Proteomics Bioinf. 13, 278–289. doi: 10.1016/j.gpb.2015.08.002 PMC4678779 26542840

[B64] Rynkiewicz E. C. Hemmerich C. Rusch D. B. Fuqua C. Clay K. (2015). Concordance of bacterial communities of two tick species and blood of their shared rodent host. Mol. Ecol. 24, 2566–2579. doi: 10.1111/mec.2015.24.issue-10 25847197

[B65] Salter S. J. Cox M. J. Turek E. M. Calus S. T. Cookson W. O. Moffatt M. F.. (2014). Reagent and laboratory contamination can critically impact sequence-based microbiome analyses. BMC Biol. 12, 87. doi: 10.1186/s12915-014-0087-z 25387460 PMC4228153

[B66] Segerman J. (1995). Siphonaptera of Southern Africa: Handbook for the identification of Fleas (Johannesburg, South Africa: Publications of the South African Institute for Medical Research).

[B67] Serratrice J. Rolain J. M. Granel B. Ene N. Conrath J. Avierinos J. F.. (2003). Occlusion bilatérale des branches de l’artère centrale de la rétine révélant une infection à *Bartonella grahamii* . Rev. Med. Interne 24, 629–630. doi: 10.1016/S0248-8663(03)00224-8 12951187

[B68] She R. Anglewicz C. Jerke K. Relich R. Glazier M. Filkins L.. (2023). *Brucella* and *Ochrobactrum* taxonomic updates for laboratories. Available online at: https://asm.org/getmedia/ae7fc7c3-0281-43aa-9143-3c9dae22a85e/Brucella-and-Ochrobactrum-Taxonomic-Updates-for-Laboratories.pdf?ext=.pdf. (Accessed December, 18, 2024).

[B69] Simpson G. J. G. Quan V. Frean J. Knobel D. L. Rossouw J. Weyer J.. (2018). Prevalence of selected zoonotic diseases and risk factors at a human-wildlife-livestock interface in Mpumalanga Province, South Africa. Vector Borne Zoonotic Dis. 18, 303–310. doi: 10.1089/vbz.2017.2158 29664701

[B70] Skinner J. D. Chimimba C. T. (2005). The mammals of the southern African sub-region (Cambridge University Press).

[B71] Stuart C. Stuart M. (2001). Stuart’s field guide to mammals of southern Africa. (South Africa: Struik Nature).

[B72] Tamura K. Stecher G. Kumar S. (2021). MEGA11: Molecular Evolutionary genetics analysis version 11. Mol. Biol. Evol. 38, 3022–3027. doi: 10.1093/molbev/msab120 33892491 PMC8233496

[B73] Telfer S. Clough H. E. Birtles L. R. Bennett M. Carslake D. Helyar S.. (2007). Ecological differences and coexistence in a guild of microparasites: *Bartonella* in wild rodents. Ecology 88, 1841–1849. doi: 10.1890/06-1004.1 17645030

[B74] Till W. M. (1963). Ethiopian mites of the genus *Androlaelaps* Berlese s. lat. (Acari: Mesostigmata). Bull. Br. Mus. Nat. Hist. Zool. 10, 1–104. doi: 10.5962/bhl.part.20524

[B75] Tipton V. J. Altman R. M. Kennan C. M. (1966). “Mites of the subfamily laelaptinae in Panama (Acarina: Laelaptidae),” in Ectoparasites of Panama. (Chicago: Field Museum of Natural History), 23–81.

[B76] Trataris A. N. Rossouw J. Arntzen L. Karstaedt A. Frean J. (2012). *Bartonella* spp. in human and animal populations in Gauteng, South Africa, from 2007 to 2009. Onderstepoort J. Vet. Res. 79, 452. doi: 10.4102/ojvr.v79i2.452 23327372

[B77] Vanderburg S. Rubach M. P. Halliday J. E. B. Cleaveland S. Reddy E. A. Crump J. A. (2014). Epidemiology of *Coxiella burnetii* infection in Africa: A one health systematic review. PloS Negl. Trop. Dis. 8, e2787. doi: 10.1371/journal.pntd.0002787 24722554 PMC3983093

[B78] Van Heerden J. Mills M. G. Van Vuuren M. J. Kelly P. J. Dreyer M. J. (1995). An investigation into the health status and diseases of wild dogs (*Lycaon pictus*) in the Kruger National Park. J. S. Afr. Vet. Assoc. 66, 18–27. Available online at: https://pubmed.ncbi.nlm.nih.gov/7629782/ 7629782

[B79] Walker A. (2000). The genus *Rhipicephalus* (Acari, Ixodidae): A guide to the brown ticks of the world; Jane B. Walker, James E. Keirans and Ivan G. Horak. Trop. Anim. Health Prod. 32, 417–418. doi: 10.1023/A:1005237804239

[B80] Woolhouse M. E. Gowtage-Sequeria S. (2005). Host range and emerging and reemerging pathogens. Emerg. Infect. Dis. 11, 1842–1847. doi: 10.3201/eid1112.050997 16485468 PMC3367654

[B81] Wu D. Yoshikawa Y. Kawamori F. Ikegaya A. Ohtake M. Ohashi M.. (2015). A molecular and serological survey of Rickettsiales bacteria in wild Sika deer (*Cervus nippon nippon*) in Shizuoka Prefecture, Japan: high prevalence of *Anaplasma* species. Jpn. J. Infect. Dis. 68, 434–437. doi: 10.7883/yoken.JJID.2015.003 25971318

[B82] Ying B. Kosoy M. Y. Maupin G. O. Tsuchiya K. R. Gage K. L. (2002). Genetic and ecologic characteristics of *Bartonella* communities in rodents in southern China. Am. J. Trop. Med. Hyg. 66, 622–627. doi: 10.4269/ajtmh.2002.66.622 12201602

