# Supplementary material for: Bacterial blood microbiome of Mastomys rodents: implications for disease spill-over at the animal-human interface within the Bushbuckridge-East community, South Africa
Source: Front Cell Infect Microbiol. 2025 Feb 3;15:1520086. doi: 10.3389/fcimb.2025.1520086 (PMC11830667; doi:10.3389/fcimb.2025.1520086)
Supplement: Supplementary file 1 [file Table1.docx]

**Table S1:** Barcode primers used for PCR amplification of the 16S rRNA gene.

| Name | Code | *Multiplex identifier (MID) sequence | ^†^Primer |
| --- | --- | --- | --- |
| MID1-27F | F1 | CGT ATC GCC TCC CTC GCG CCA TCAG ACG AGT GCGT | 27F |
| MID2-27F | F2 | CGT ATC GCC TCC CTC GCG CCA TCAG ACG CTC GACA | 27F |
| MID3-27F | F3 | CGT ATC GCC TCC CTC GCG CCA TCAG AGA CGC ACTC | 27F |
| MID4-27F | F4 | CGT ATC GCC TCC CTC GCG CCA TCAG AGC ACT GTAG | 27F |
| MID5-27F | F5 | CGT ATC GCC TCC CTC GCG CCA TCAG TCA GAC ACGA | 27F |
| MID1-1435R | R1 | CTA TGC GCC TTG CCA GCC CGC TCAG ACG AGT GCGT | 1435R |
| MID2-1435R | R2 | CTA TGC GCC TTG CCA GCC CGC TCAG ACG CTC GACA | 1435R |
| MID3-1435R | R3 | CTA TGC GCC TTG CCA GCC CGC TCAG AGA CGC ACTC | 1435R |
| MID4-1435R | R4 | CTA TGC GCC TTG CCA GCC CGC TCAG AGC ACT GTAG | 1435R |
| MID5-1435R | R5 | CTA TGC GCC TTG CCA GCC CGC TCAG ATC AGA CACG | 1435R |

*red font= Adaptor sequence, blue font= Key sequence, green font= MID sequence.

^†^27F = 5’-AGA GTT TGA TCM TGG CTC AGA ACG; 1435R= 5’-CGA TTA CTA GCG ATT CCR RCT TCA

**Table S2:** Information for rodent samples.

| Sample number | Origin of rodent | Habitat | Rodent species^a^ | Sex | Barcode primer ID^b^ |
| --- | --- | --- | --- | --- | --- |
| R19 | Gottenburg | urban/peri-urban | *M. natalensis* | male | F2R5 |
| R20 | Gottenburg | urban/peri-urban | *M. coucha* | male | F3R4 |
| R29 | Gottenburg | urban/peri-urban | *M. natalensis* | male | F3R1 |
| R30 | Gottenburg | urban/peri-urban | *M. natalensis* | male | F3R2 |
| R31 | Gottenburg | urban/peri-urban | *M. natalensis* | female | F3R3 |
| R171 | Gottenburg | urban/peri-urban | *M. natalensis* | female | F1R2 |
| R172 | Gottenburg | urban/peri-urban | *M. natalensis* | female | F1R3 |
| R74 | Hlalakahle | urban/peri-urban | *M. natalensis* | male | F4R5 |
| R75 | Hlalakahle | urban/peri-urban | *M. natalensis* | male | F5R1 |
| R78 | Hlalakahle | urban/peri-urban | *M. natalensis* | female | F5R2 |
| R95 | Hlalakahle | urban/peri-urban | *M. natalensis* | female | F4R4 |
| R98 | Hlalakahle | urban/peri-urban | *M. natalensis* | male | F1R5 |
| R99 | Hlalakahle | urban/peri-urban | *M. natalensis* | female | F5R3 |
| R2 | Tlhavekisa | communal rangeland | *M. coucha* | female | FIR1 |
| R5 | Tlhavekisa | communal rangeland | *M. coucha* | male | F2R2 |
| R6 | Tlhavekisa | communal rangeland | *M. coucha* | male | F1R4 |
| R11 | Tlhavekisa | communal rangeland | *M. coucha* | female | F2R3 |
| R12 | Tlhavekisa | communal rangeland | *M. coucha* | female | F2R4 |
| R84 | Tlhavekisa | communal rangeland | *M. natalensis* | female | F2R1 |
| R21 | Manyeleti | protected area | *M. natalensis* | female | F3R5 |
| R53 | Manyeleti | protected area | *Steatomys sp.* | female | F4R2 |
| R61 | Manyeleti | protected area | *M. coucha* | male | F4R3 |
| R159 | Manyeleti | protected area | *M. coucha* | male | F5R4 |
| R177 | Manyeleti | protected area | *M. coucha* | female | F5R5 |
| R179 | Manyeleti | protected area | *M. coucha* | male | F4R1 |

^a^ Species identification was confirmed by molecular typing of the cytochrome c oxidase gene and the cytochrome *b* gene.

^b^ Sample-specific primer combination used to amplify each sample to construct the library for circular consensus sequencing (CCS); See Table 1 for detailed primer information.

**TABLE S3**. Sequence read archive (SRA) accession numbers of bacterial sequences obtained from rodents.

| Accession number | Sample number | Rodent species |
| --- | --- | --- |
| SRX5967121 | R172 | *Mastomys natalensis* |
| SRX5967122 | R21 | *Mastomys natalensis* |
| SRX5967123 | R29 | *Mastomys natalensis* |
| SRX5967124 | R30 | *Mastomys natalensis* |
| SRX5967125 | R31 | *Mastomys natalensis* |
| SRX5967126 | R171 | *Mastomys natalensis* |
| SRX5967127 | R98 | *Mastomys natalensis* |
| SRX5967128 | R99 | *Mastomys natalensis* |
| SRX5967129 | R19 | *Mastomys natalensis* |
| SRX5967130 | R20 | *Mastomys coucha* |
| SRX5967131 | R179 | *Mastomys coucha* |
| SRX5967132 | R61 | *Mastomys coucha* |
| SRX5967133 | R53 | *Steatomys* |
| SRX5967134 | R177 | *Mastomys coucha* |
| SRX5967135 | R159 | *Mastomys coucha* |
| SRX5967136 | R74 | *Mastomys natalensis* |
| SRX5967137 | R75 | *Mastomys natalensis* |
| SRX5967138 | R12 | *Mastomys coucha* |
| SRX5967139 | R84 | *Mastomys natalensis* |
| SRX5967140 | R6 | *Mastomys coucha* |
| SRX5967141 | R11 | *Mastomys coucha* |
| SRX5967142 | R2 | *Mastomys coucha* |
| SRX5967143 | R5 | *Mastomys coucha* |
| SRX5967144 | R78 | *Mastomys natalensis* |
| SRX5967145 | R95 | *Mastomys natalensis* |

**TABLE S4.** Genbank accession numbers of sequences used for the phylogenetic analysis of the 16S rRNA gene of *Bartonella* spp.

| Accession number | Organism | Strain/Country | Host |
| --- | --- | --- | --- |
| NR104902 | *Bartonella vinsonii* subsp. *arupensis* | OK 94-513/Sweden | Culture |
| DQ228135 | *Bartonella vinsonii* subsp. *berkhofii* | Q64SHD/China | Dog |
| AJ250247 | *Bartonella quintana* | Italy | *Pediculus humanus corporis* |
| NR074335 | *Bartonella henselae* | Houston-1/USA | Cat |
| NR178638 | *Bartonella mastomydis* | 008/Senegal | *Mastomys erythroleucus* |
| EU111756 | *Bartonella queenslandensis* | AUST/NH8/Australia | *Melomys* sp. |
| NR074354 | *Bartonella tribocorum* | IBS 506/France | *Rattus* sp. |
| NR115255 | *Bartonella rattimassiliensis* | 15908/France | *Rattus norvegicus* |
| CP001562 | *Bartonella grahamii* | as4aup/Sweden | *Apodemus flavicollis* |
| NR116175 | *Bartonella rattaustraliani* | AUST/NH4/Australia | *Rattus tunneyi* |
| NR025889 | *Bartonella elizabethae* | F9251/USA | Human |
| NR115254 | *Bartonella phoceensis* | 16120/France | *Rattus norvegicus* |
| AB602532 | *Bartonella pachyuromydis* | FN18-1/Netherlands | *Pachyuromys duprasi* |
| AY993936 | *Bartonella* sp. | RF255YX /China | *Rattus tanezumi flavipectus* |
| NR_074243 | *Ochrobactrum anthropi* | ATCC 49188/USA | Culture |


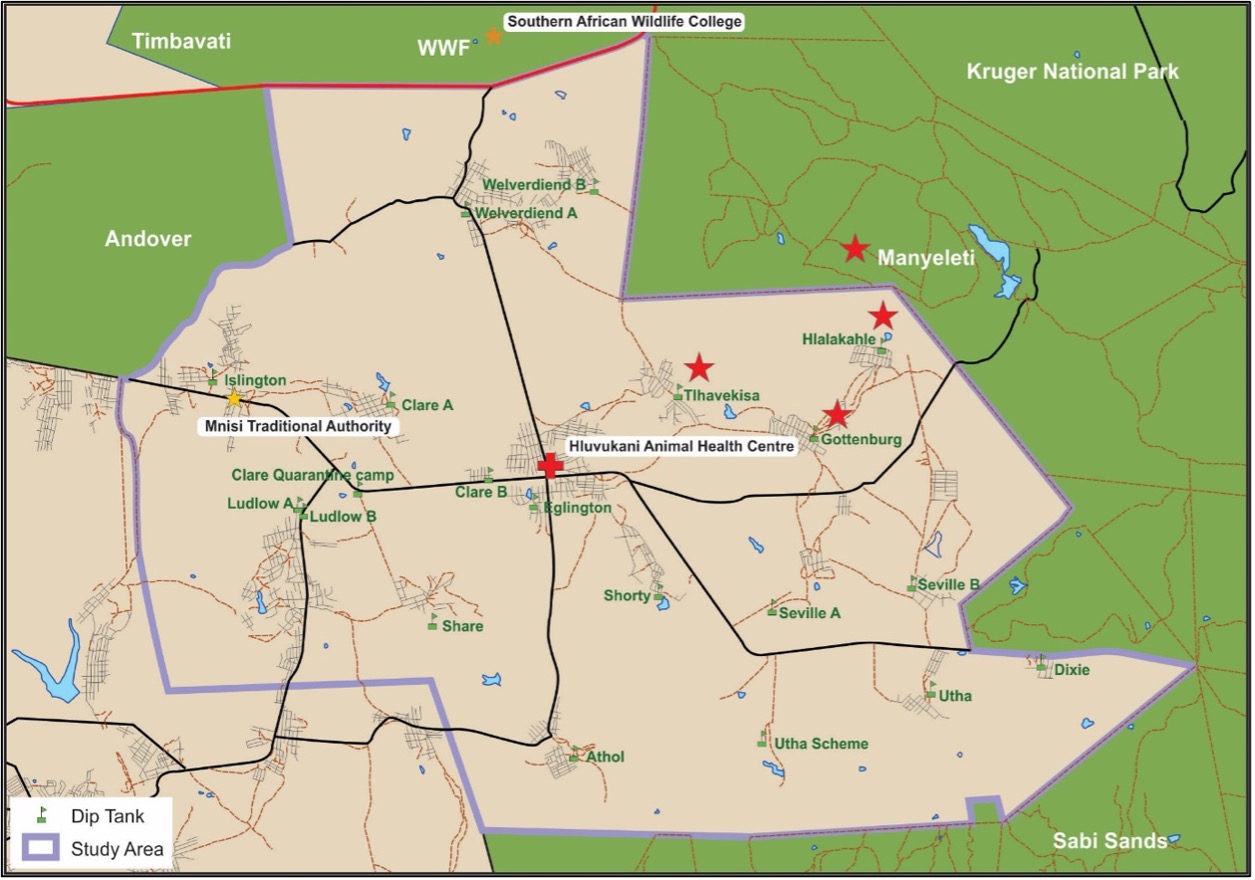


**Figure S1.** Map of the study area in Bushbuckridge-East Community, Mpumalanga Province, South Africa. Red stars show sites of rodent capture. Gottenburg and Hlalakahle are urban or peri-urban areas, while Tlhavekisa is in the communal rangelands. Manyeleti is a protected wildlife reserve. Dark green represents protected areas where wildlife roam freely.


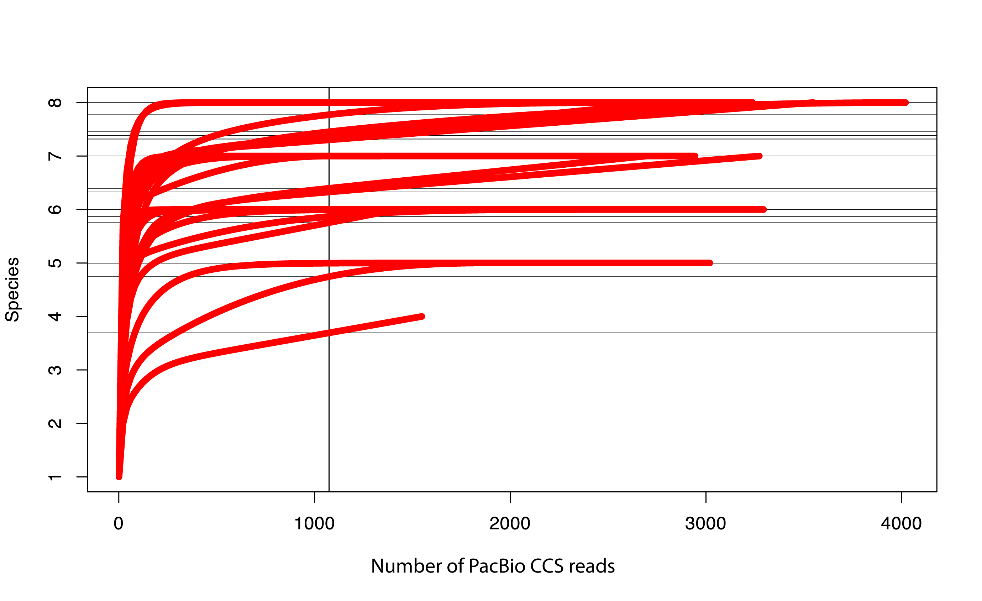


**Figure S2.** Rarefaction curves of bacterial species (OTUs) identified in rodent blood samples. The number of bacterial species detected in rodent blood samples was plotted as a function of read depth. The vertical line in the plot indicates the value where rarefaction criterion was satisfied.


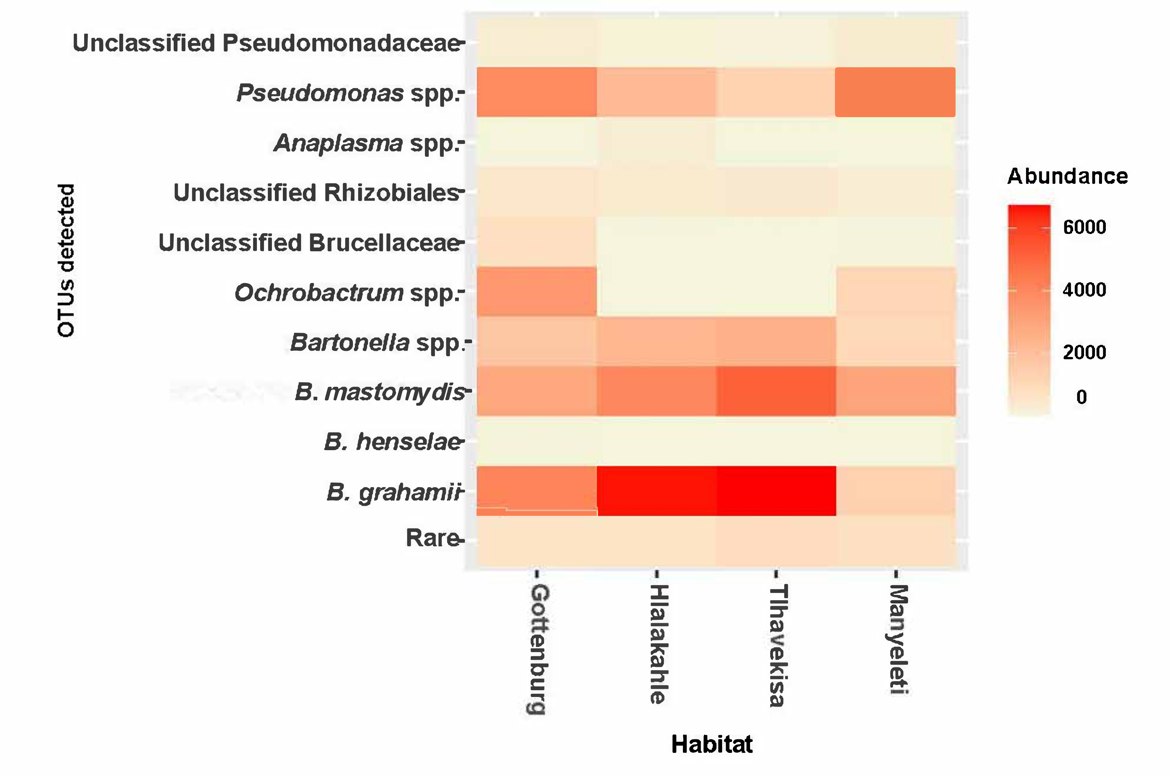


**FIGURE S3.** Heatmap showing the abundance of operational taxonomic units (OTUs) detected in rodent samples from the four habitats. Habitat names are shown on the X axis and bacterial sequences detected in rodent blood are depicted on the Y axis.


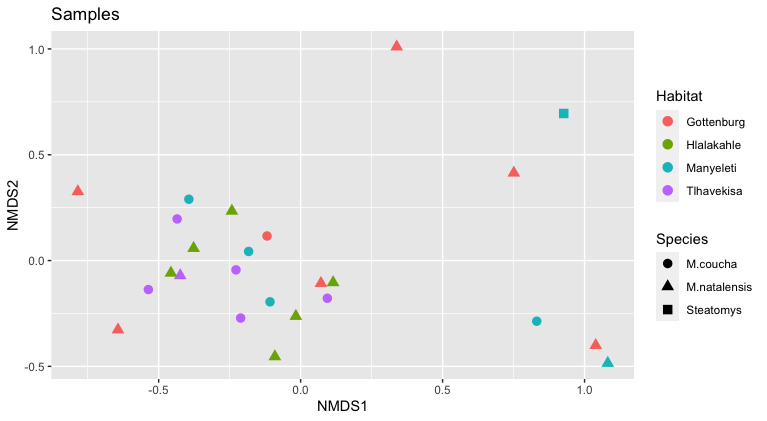


**FIGURE S4.** NMDS plot of generalized ordination distances between rodent samples. Red shapes indicate rodents captured from Gottenburg, green shapes – rodents from Hlalakahle, blue shapes – rodents from Manyeleti and purple shapes – rodents from Tlhavekisa. *Mastomys coucha* are represented by the circles, *M. natalensis* by the triangles and *Steatomys* sp. by the square.


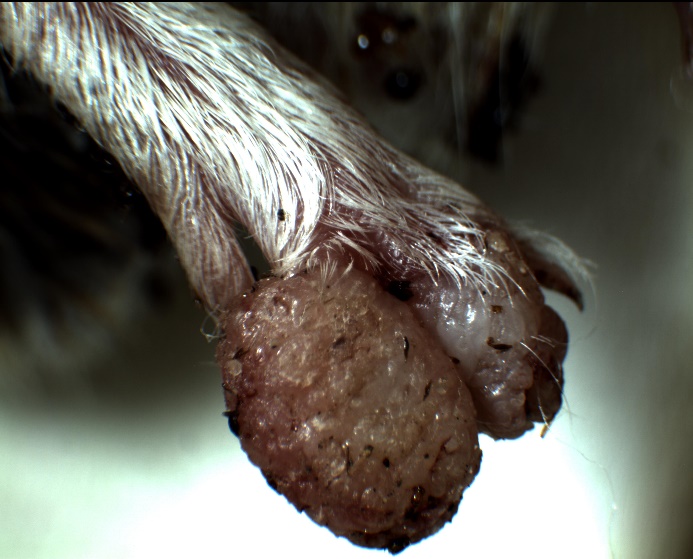

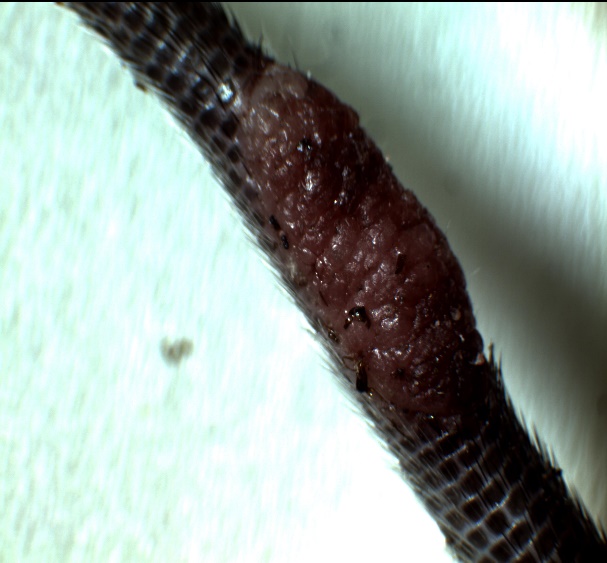


**FIGURE S5.** Ulcerative pododermatitis lesions in foot and tail of some rodents in this study. Previous research indicates such lesions are associated with *Pseudomonas* spp. infection.

**R scripts for microbiome analysis**

R studio was used to import sample data, and the vegan package was used to calculate the mean species diversity of bacteria in all rodents from the different habitat areas using rarefaction curves. FactoMineR package was used to conduct Principal Component Analysis (PCA) to quantify the compositional similarity or dissimilarity of the bacterial population in the blood of the rodents across the habitat areas. Statistical test of PerMANOVA (Permutational ANOVA) was conducted using the adonis function in the vegan package. Nonmetric multidimensional scaling (NMDS) ordination to compare samples based on their rarefied bacterial populations was done using the phyloseq package.

**Rarefaction curves**

>setwd("C:/Users/User/Desktop")

>rodent <- read.table("OtuRodent.txt", header=T, row.names=1, sep="\t")

>library(vegan)

>raremax <- min(rowSums(rodent))

>col <- c("purple")

>lty <- c("solid")

>lwd <- c("4")

>pars <- expand.grid(col = col, lty = lty, lwd = lwd, stringsAsFactors = FALSE)

>head(pars)

>out <- with(pars, rarecurve(rodent, step = 20, sample = raremax, col = col, lty = lty, lwd =lwd, label = FALSE))

**Principal component analysis (FactoMineR)**

>rodent <- read.table("rodent.txt", header=T, row.names=1, sep="\t")

>library(FactoMineR)

>library (factoextra)

>res <- PCA(rodent[,1:11])

>summary(res)

>res <- PCA(rodent, quali.sup=12)

>plot(res, cex=0.8, habillage="Habitat")

>fviz_pca_ind(res, cex=1.2, label="none", habillage="Habitat")

**Description of the dimensions**

>dimdesc(res)

**PerMANOVA (Vegan)**

>rm <- read.table("Rodentmeta.txt", header=T, row.names=1, sep="\t")

>library(vegan)

> R.dist=vegdist(rm, distance="bray")

>adonis(R.dist ~ Gottenburg*Manyeleti, data =rm, permutations = 1000)

>adonis(R.dist ~ Manyeleti*Tlhavekisa, data=rm, permutations = 1000)

>adonis(R.dist ~ Hlalakahle*Gottenburg, data=rm, permutations = 1000)

>adonis(R.dist ~ Gottenburg*Hlalakahle, data=rm, permutations = 1000)

>adonis(R.dist ~ Manyeleti*Hlalakahle, data=rm, permutations = 1000)

>adonis(R.dist ~ Gottenburg*Tlhavekisa, data=rm, permutations = 1000)

>adonis(R.dist ~ Hlalakahle*Tlhavekisa, data=rm, permutations = 1000)

**Ordination Script (Phyloseq)**

>library("phyloseq")

>library("ggplot2")

>library("plyr")

>library("vegan")

>library(microbiome)

>otu_mat <- read.table("Rodent1.txt", header=T, row.names=1, sep="\t")

>tax_mat <- read.table("TaxRodent.txt", header=T, row.names=1, sep="\t")

>samples_df<- read.table("SampleRodent.txt", header=T, row.names=1, sep="\t", check.names = TRUE, stringsAsFactors = TRUE)

>row.names(otu_mat) <- otu_mat$otu

>row.names(tax_mat) <- tax_mat$otu

>row.names(samples_df) <- samples_df$sample

>otu_mat <- as.matrix(otu_mat)

>tax_mat <- as.matrix(tax_mat)

>OTU = otu_table(otu_mat, taxa_are_rows = TRUE)

>TAX = tax_table(tax_mat)

>rownames(samples_df)=colnames(otu_mat)

>samples=as.data.frame(samples_df)

>mysamples=sample_data(as.data.frame(samples_df))

>set2 <- phyloseq(OTU, TAX, mysamples)

>set2

>sample_names(set2)

>rank_names(set2)

>sample_variables(set2)

>plot_bar(set2, fill = "Genus")

>plot_bar(set2, fill = "Species")

>plot_heatmap(set2, method = "NMDS", distance = "bray")

>plot_heatmap(set2, method = "MDS", distance = "(A+B-2*J)/(A+B-J)",

taxa.label = "Genus", taxa.order = "Genus",

trans=NULL, low="beige", high="red", na.value="beige")

>plot_heatmap(set2, method = "MDS", distance = "(A+B-2*J)/(A+B-J)",

taxa.label = "Species", taxa.order = "Species",

trans=NULL, low="beige", high="red", na.value="beige")

>plot_heatmap(set2, method = "NMDS", distance = "bray", taxa.label = "Species", taxa.order = "Species",

low="beige", high="red", na.value="beige")

>dist_methods <- unlist(distanceMethodList)

>print(dist_methods)

>plot_richness(set2, measures=c("Chao1", "Shannon"))

>plot_richness(set2, measures=c("Chao1", "Shannon"), x="Species", color="Habitat")

>set2.ord <- ordinate(set2, "NMDS", "bray")

>plot_ordination(set2, set2.ord, type="taxa", color="Species", shape= "Genus",

title="OTUs")

>plot_ordination(set2, set2.ord, type="samples", color="Habitat",

shape="Species", title="Samples") + geom_point(size=3)

**
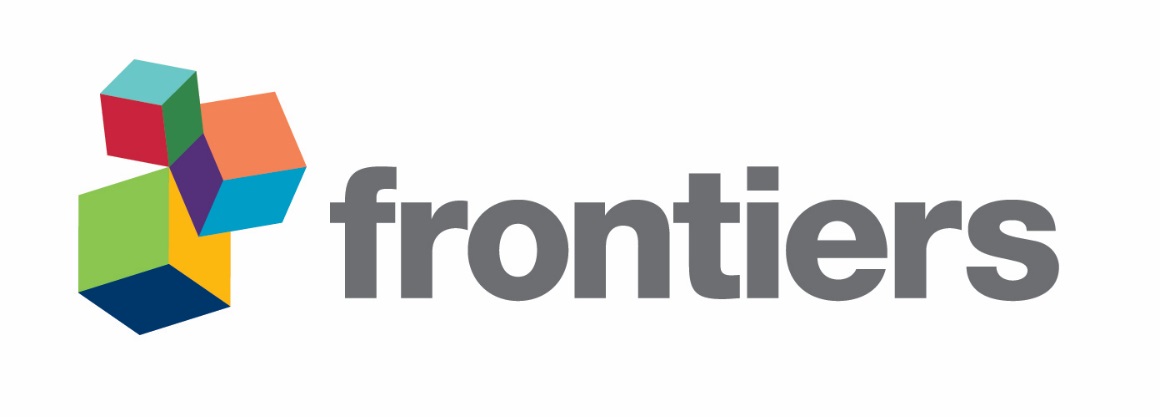
**
